# Supplementary material for: Atlas of lysine acetylation in the mouse
Source: bioRxiv. 2026 Jan 10:2026.01.09.698739. Preprint. [Version 1] doi: 10.64898/2026.01.09.698739 (PMC12803184; doi:10.64898/2026.01.09.698739)
Supplement: 1 [file NIHPP2026.01.09.698739V1-supplement-1.pdf]

bioRxiv preprint doi: https://doi.org/10.64898/2026.01.09.698739; this version posted January 10, 2026. The copyright holder for this preprint (which was not certified by peer review) is the author/funder, who has granted bioRxiv a license to display the preprint in perpetuity. It is made available under aCC-BY 4.0 International license.

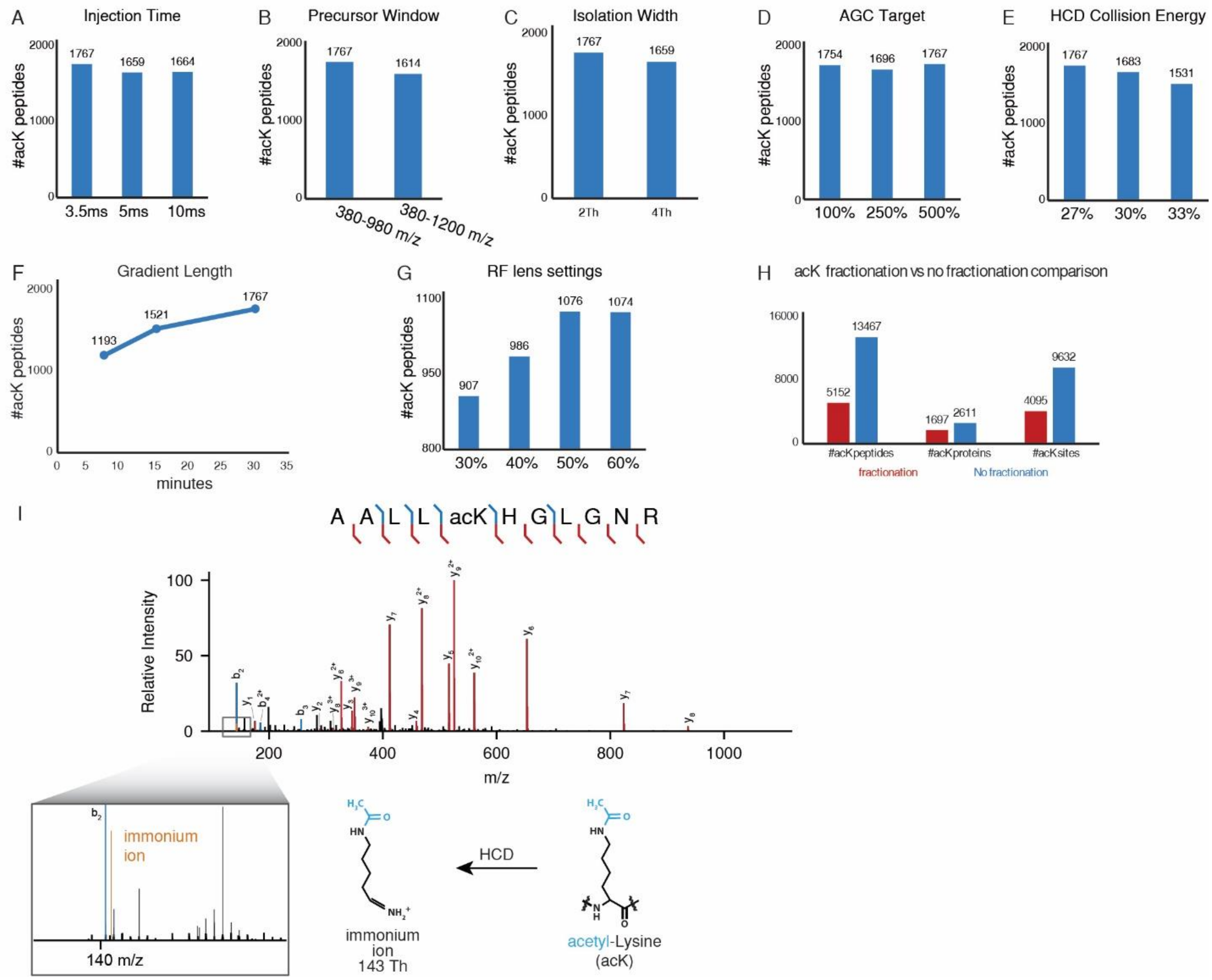

Supplementary Figure 1. LC-MS parameter sweeping and acetylpeptide protocol optimization. **A-G** LC-MS parameter sweeping. All parameters tested via 250ng male mouse liver acetylpeptide injection with n=1 injection per parameter setting. **H** Comparison between analysis of single batch of enriched acetylpeptides from male mouse liver with our without high-pH fractionation. Fractionation was performed on an Agilent 1260 Infinity BioInert LC with an automated fraction collector over a 20-minute method with a Waters XBridge, Peptide BEH C18, 3.5 µm, 130 Å, 4.6 mm x 150 mm column at 0.8 mL/min as previously described[17]. **I** Tandem mass spectrum of representative acetylpeptide exhibiting inclusion of the lysine acetylation reporter ion, the immonium ion.

bioRxiv preprint doi: https://doi.org/10.64898/2026.01.09.698739; this version posted January 10, 2026. The copyright holder for this preprint (which was not certified by peer review) is the author/funder, who has granted bioRxiv a license to display the preprint in perpetuity. It is made available under aCC-BY 4.0 International license.

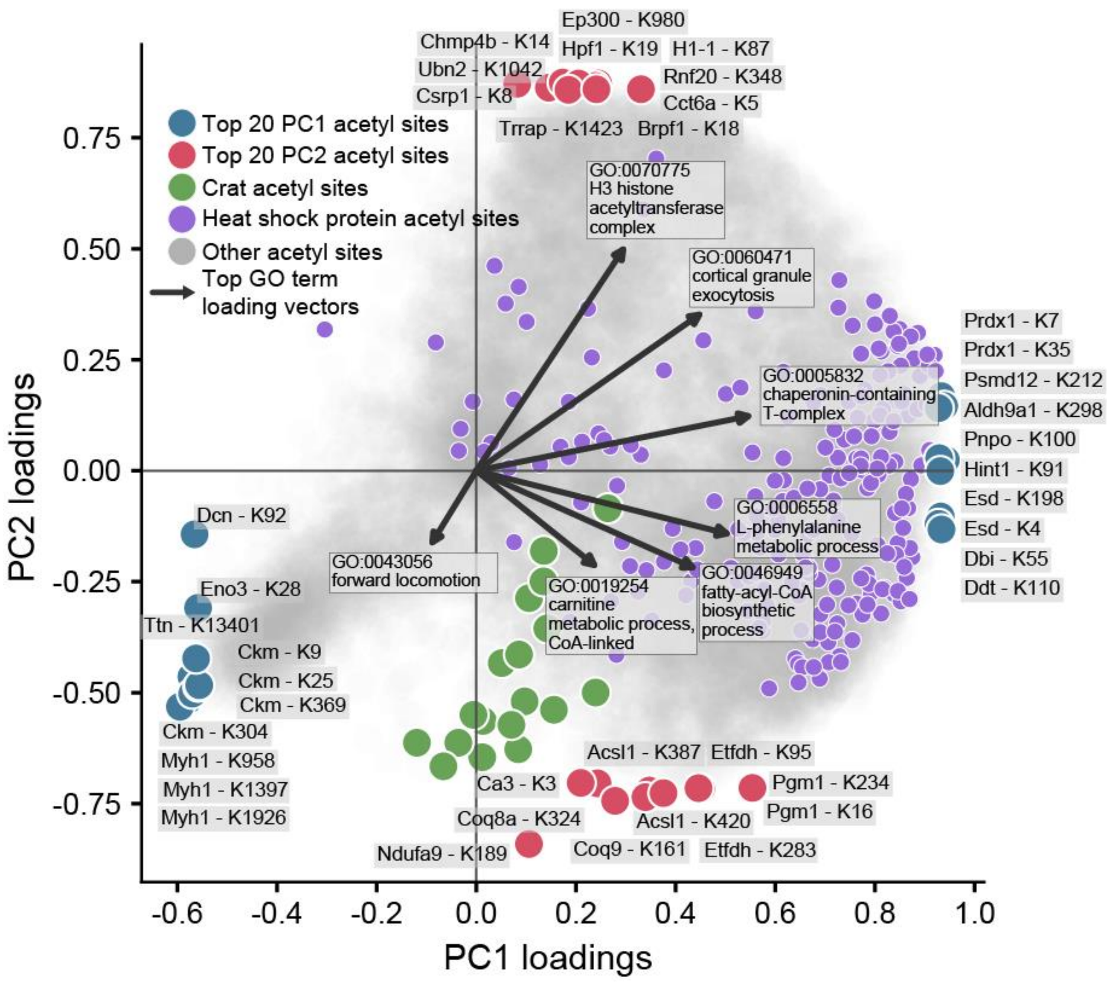

Supplementary Figure 2. PCA loadings and top loadings drivers. Loadings graph detailing major drivers of Figure 3A PCA.

bioRxiv preprint doi: https://doi.org/10.64898/2026.01.09.698739; this version posted January 10, 2026. The copyright holder for this preprint (which was not certified by peer review) is the author/funder, who has granted bioRxiv a license to display the preprint in perpetuity. It is made available under aCC-BY 4.0 International license.

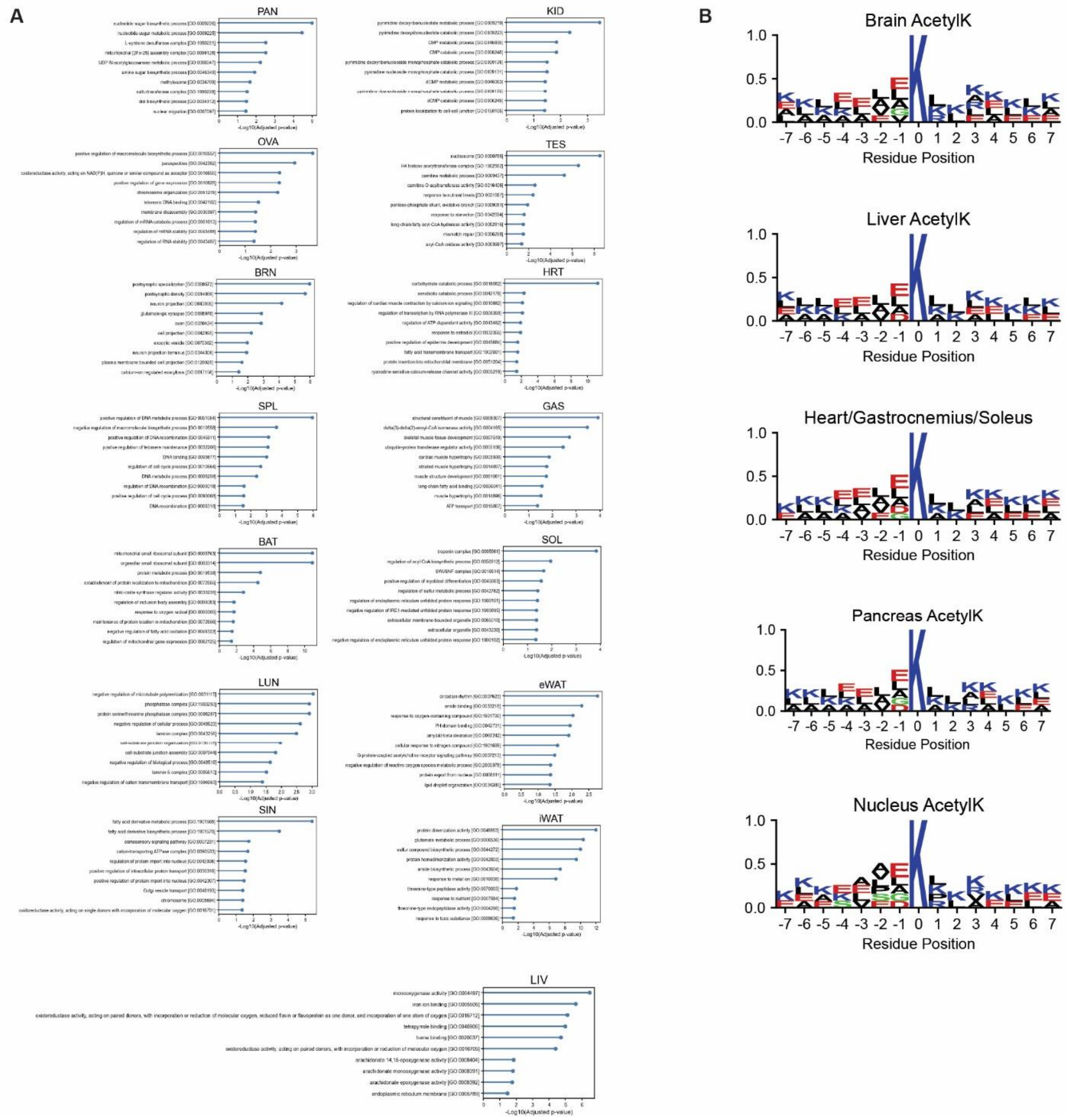

Supplementary Figure 3. Tissue Specific GO term enrichment and selected sequence motifs. A Top 10 GO terms for acetyl-lysine sites found uniquely enriched in each individual tissue. **B** Selected sequence motifs for brain, liver, muscle, and pancreas acetyl sites as well as nuclear-localized acetyl sites.

bioRxiv preprint doi: https://doi.org/10.64898/2026.01.09.698739; this version posted January 10, 2026. The copyright holder for this preprint (which was not certified by peer review) is the author/funder, who has granted bioRxiv a license to display the preprint in perpetuity. It is made available under aCC-BY 4.0 International license.

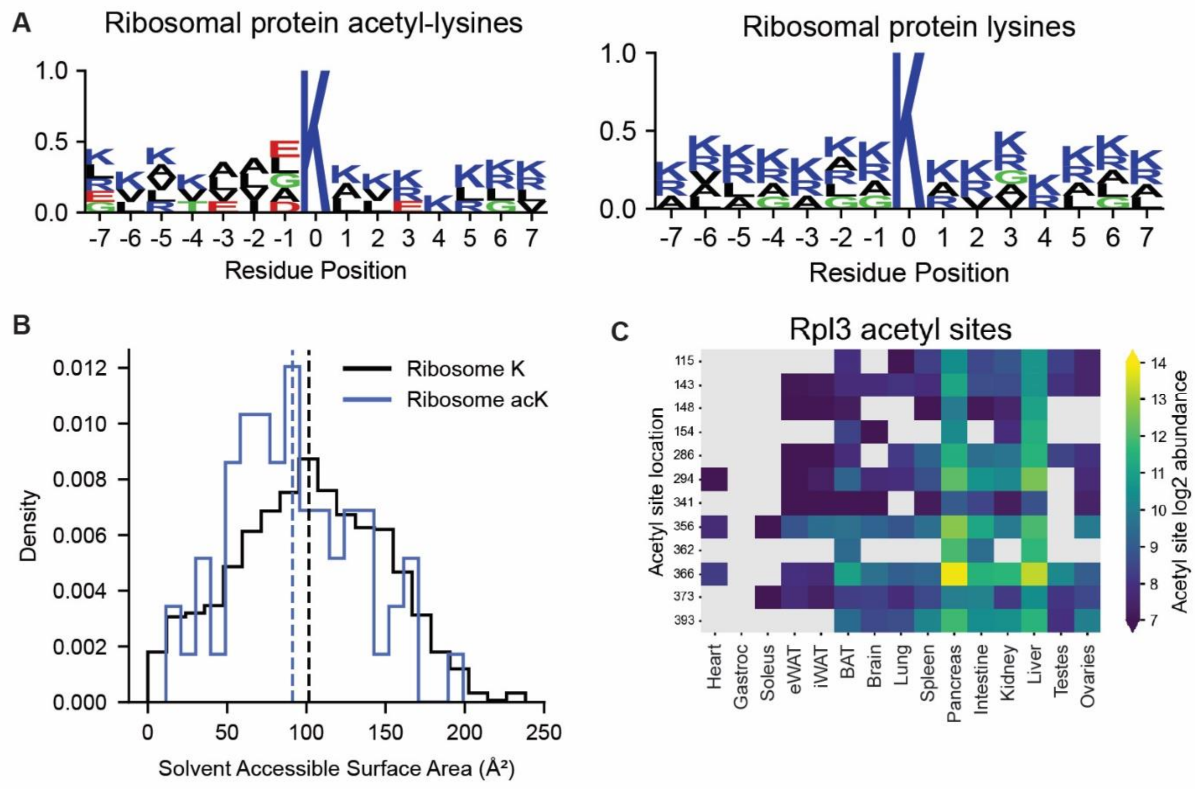

Supplementary Figure 4. Ribosomal acetylation. A Sequence motif of all detected ribosomal protein acetyl-lysine sites. **B** Solvent accessible surface area (SASA) distribution of all ribosomal lysines and all observed ribosomal acetyl-lysines. **C** Clustered heatmap detailing all detected acetyl-lysines found on Rpl3 across all tissues, sex agnostic.

bioRxiv preprint doi: https://doi.org/10.64898/2026.01.09.698739; this version posted January 10, 2026. The copyright holder for this preprint (which was not certified by peer review) is the author/funder, who has granted bioRxiv a license to display the preprint in perpetuity. It is made available under aCC-BY 4.0 International license.

Mice	blood glucose (mg/dL)
Male 1	174
Male 2	216
Male 3	183
Female 1	144
Female 2	145
Female 3	169

Supplementary Table 1. Blood glucose levels of mice at time of tissue harvest.

bioRxiv preprint doi: https://doi.org/10.64898/2026.01.09.698739; this version posted January 10, 2026. The copyright holder for this preprint (which was not certified by peer review) is the author/funder, who has granted bioRxiv a license to display the preprint in perpetuity. It is made available under aCC-BY 4.0 International license.

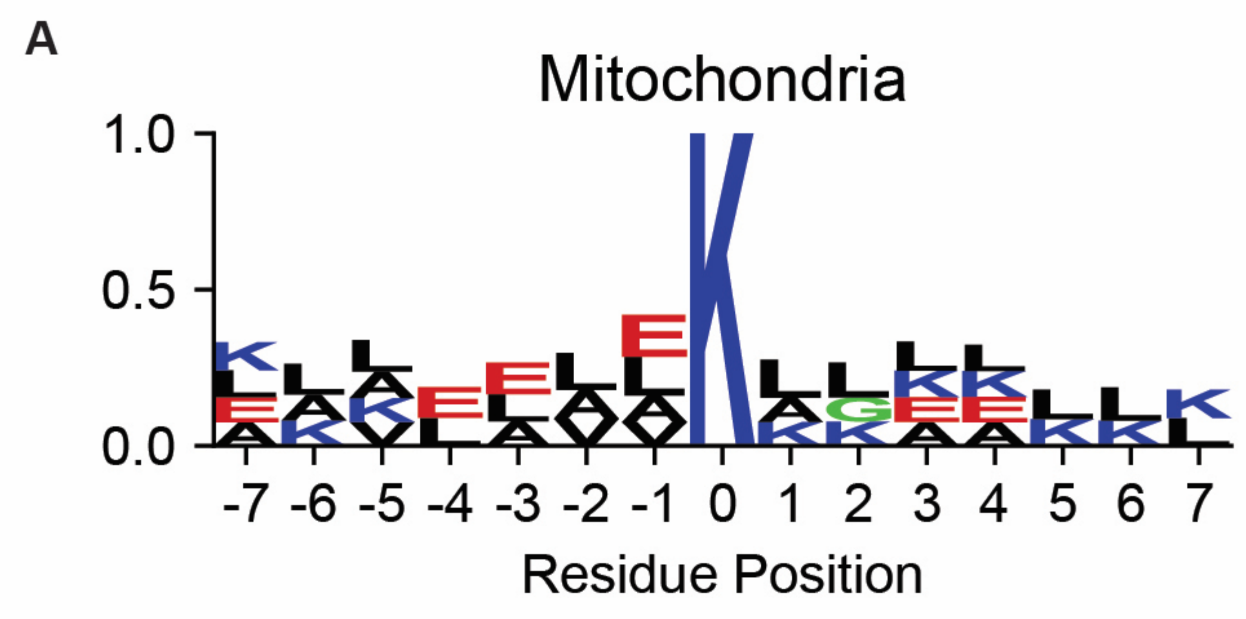

Supplementary Figure 5. Mitochondrial protein sequence motif.

bioRxiv preprint doi: https://doi.org/10.64898/2026.01.09.698739; this version posted January 10, 2026. The copyright holder for this preprint (which was not certified by peer review) is the author/funder, who has granted bioRxiv a license to display the preprint in perpetuity. It is made available under aCC-BY 4.0 International license.

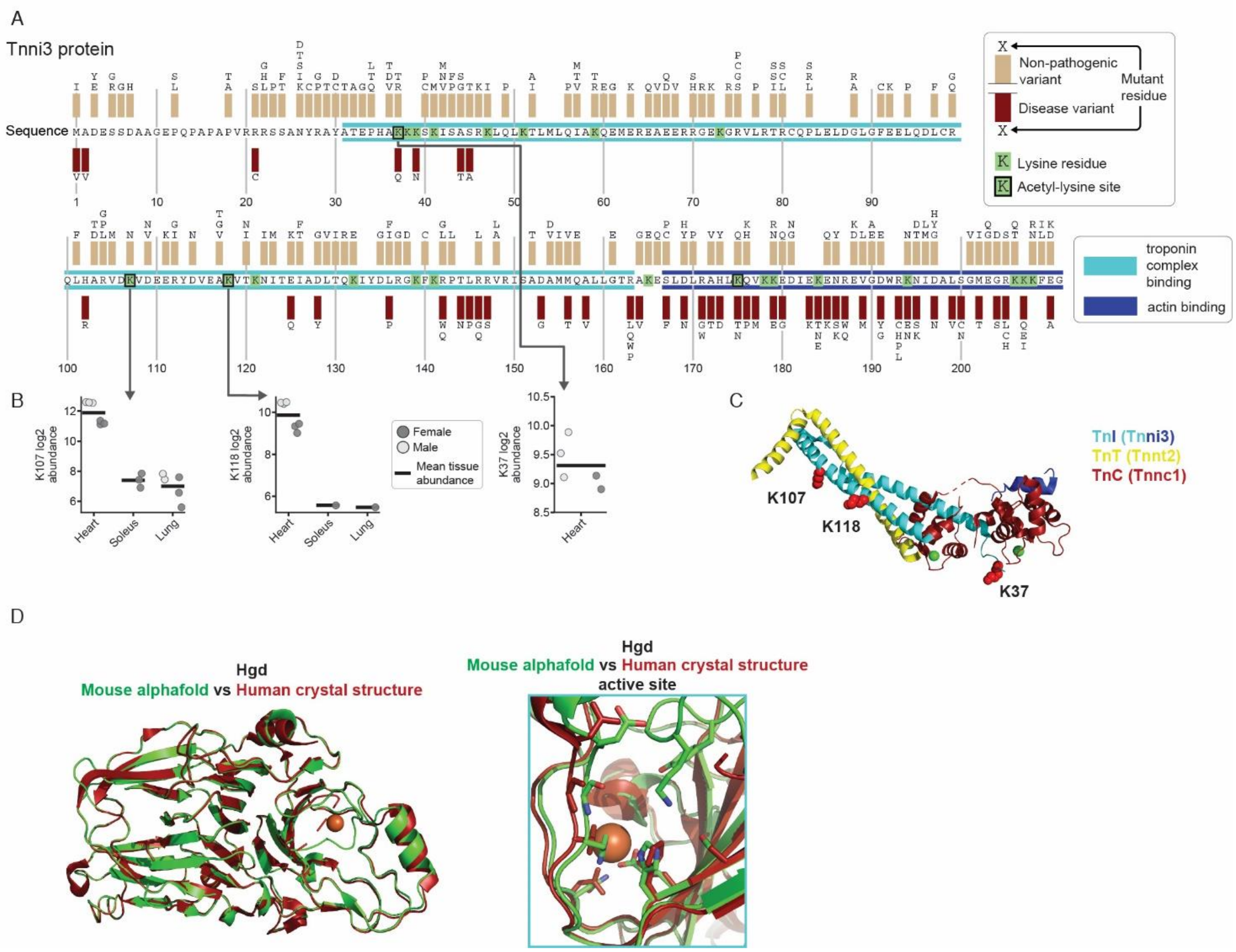

Supplementary Figure 6. Full H2M acK analysis and Tnni3 acetylation. A Sequence of human Tnni3 with troponin complex binding domain highlighted in cyan and the actin binding site highlighted in dark blue. All reported SNPs annotated. **B** Abundance of selected acetyl-sites in mouse tissues. C Structure of human tropomyosin complex crystal structure (RSCB PDB ID: 1J1E). Detected lysine acetylation sites colored in red spheres. Tnni3 colored in cyan and dark blue (same as above). Tnnt2 colored in yellow. Tnnc1 colored in red. D Alignment of red human Hgd crystal structure (RSCB PDB ID: 1EY2) and green mouse Hgd predicted structure (EMBL:EBI AF-O09173-F1-v6) and active site.
